# A Range Ambiguity Suppression Processing Method for Spaceborne SAR with Up and Down Chirp Modulation

**DOI:** 10.3390/s18051454

**Published:** 2018-05-07

**Authors:** Xuejiao Wen, Xiaolan Qiu, Bing Han, Chibiao Ding, Bin Lei, Qi Chen

**Affiliations:** 1 Laboratory of spatial information intelligent processing system, Institute of Electronics, Chinese Academy of Sciences, Suzhou 215000, China; xjwen@mail.ie.ac.cn; 2 Institute of Electronics, Chinese Academy of Sciences, Beijing 100190, China; han_bing@mail.ie.ac.cn (B.H.); cbding@mail.ie.ac.cn (C.D.); leibin@mail.ie.ac.cn (B.L.); 3 China Center for Resources Satellite Data and Application, Beijing 100094, China; chenq_cn@163.com

**Keywords:** range ambiguity suppression, spaceborne SAR, Chinese Gaofen-3 SAR sensor

## Abstract

Range ambiguity is one of the factors which affect the SAR image quality. Alternately transmitting up and down chirp modulation pulses is one of the methods used to suppress the range ambiguity. However, the defocusing range ambiguous signal can still hold the stronger backscattering intensity than the mainlobe imaging area in some case, which has a severe impact on visual effects and subsequent applications. In this paper, a novel hybrid range ambiguity suppression method for up and down chirp modulation is proposed. The method can obtain the ambiguity area image and reduce the ambiguity signal power appropriately, by applying pulse compression using a contrary modulation rate and CFAR detecting method. The effectiveness and correctness of the approach is demonstrated by processing the archive images acquired by Chinese Gaofen-3 SAR sensor in full-polarization mode.

## 1. Introduction

In Synthetic Aperture Radar (SAR) imaging, range ambiguity is caused by the echoes of the previous and latter transmitted pulses scattered from undesired range zones [1]. The round-trip propagation time of the ambiguous signals differ from that of the desired signal by a multiple of the reciprocal pulse repetition frequency (PRF). It is more severe in spaceborne cases than airborne cases with the increase of PRF, which may dramatically affect the quality of the reconstructed image. Thus, range ambiguity suppression is a highly regarded problem in SAR signal processing.

Compared with a variety of azimuth ambiguity suppression techniques [2,3,4], the study of range ambiguity suppression mainly focused on the radar system. There are mainly two kinds of range ambiguity suppression techniques for SAR systems. One kind of ideas try to modify the way of transmitting and receiving, so as to block the receiving of the ambiguous energy, such as elevation null spacing [5], multiple elevation beams [6], azimuth phase coding(APC) [7,8], and digital beam forming on receiving which can forms an equivalent narrow beam of high antenna gain pointing to the imaging area and suppressing the ambiguity [9]. The others are algorithms which aim to disperse the peak ambiguous energy to reduce the impaction of ambiguity, for example, the finite depth of focus algorithm which automatically defocuses the range ambiguities, thereby reducing their peak levels [10,11], and more commonly modulation of successively transmitted pulses by alternating up and down chirps which spreads the ambiguities in the range direction [12,13]. This technique significantly reduces the ambiguity peaks and performs well for multi-polarization spaceborne SAR where a serious decline in the ambiguity ratio appears in cross polarization mode. With the development of new SAR systems, some new techniques have been put forward in recent years, for example, coding the transmitted pulses using an orthogonal frequency-division multiplexing (OFDM) pulse [14], frequency division segregation scheme of uniformly dividing the entire bandwidth into several smaller bands according to the ground-range resolution [15]. In order to solve the conflict between range and azimuth ambiguities in spaceborne high-resolution and wide-swath (HRWS) SAR, some new techniques based on frequency diverse array (FDA) are proposed [16,17]. Furthermore, the performances of range ambiguity suppression in multichannel ScanSAR with up and down chirp modulation have been studied in [13].

It can be seen that in order to adapt to the new mode SAR, many improvements in radar system design for range ambiguity suppression have been put forward. However, there is a lack of analysis and solutions to the problems that may exist in these system designs. Take the technique of up and down chirp modulation for example, it will show severe destructions to the image quality under certain conditions where targets appearing in the ambiguous area have a stronger backscattering intensity than the imaging targets of interest, such as ships or towns in the ambiguous area for the imaging targets like rivers or mountains. The energy of ambiguous area after SAR imaging process are still too high to reduce the ambiguity ratio which leads to line defocusing ambiguity in the image, having severe impact on visual effects and subsequent applications.

Gaofen-3 satellite is the first full-polarization SAR sensor of China, which implemented alternatively up and down chirp modulation for range ambiguity suppression. The line defocusing ambiguity problem appeared in certain images acquired in full-polarization mode. Therefore, this paper proposed a novel hybrid range ambiguity suppression method for up and down chirp modulation. The method can obtain the ambiguity area image and reduce the ambiguity signal power appropriately, by applying pulse compression using contrary modulation rate and Constant False Alarm Rate (CFAR) detecting method.

In Section 2 the effect of range ambiguity under the condition of transmitting up and down chirp modulation signals is analyzed. In Section 3 the principles of the proposed range ambiguity suppression technique are introduced. Section 4 gives the processing results of Gaofen-3 full-polarization strip mode data and conclusion is summarized in the Section 5.

## 2. Range Ambiguity Analysis by Alternatively Up and Down Chirp Modulation

**Figure 1:**
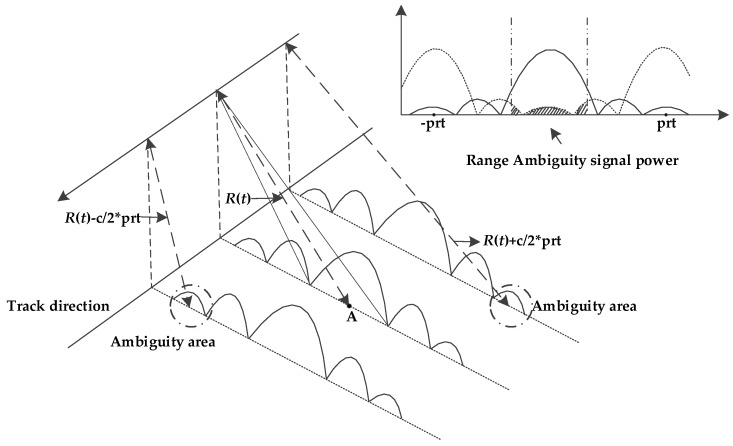
This is a figure which shows the principle of range ambiguity.

As shown in Figure 1, range ambiguous signals are echoes arrived at the wrong time and scattered from undesired range zones. Considering a target (point A in Figure 1) in the main region with range history *R*(*t*) where *t* presents the azimuth time, the echoes from previous and latter transmitted pulses will arrive as ambiguous signals with the slant range history as [18]:

(1) $$R_{n}^{\prime }(t)=R(t)+c/2\cdot n/prf(n=\pm 1,\pm 2,\pm 3\ldots )$$

where *c* is the velocity of light, *prf* is the pulse repetition frequency, *n* is the ambiguous number (negative part presents previous pulses when positive part presents latter pulses).

The signal echoes consist of mainlobe area parts and ambiguous area parts are shown below:

(2) $$s(\tau ,t)=s_{main}(\tau ,t)+\sum_{n}s_{ambiguity}(\tau ,t,n)$$

where *τ* is the range time.

Supposing the system works by sending alternately up and down chirp signals with pulse length *T_p_* and modulation rate *k_r_*, We can get the echo of ambiguous signals as:

(3) $$\begin{array}{ll} s_{ambiguity}(\tau ,t,n) & =\sigma_{n}\cdot W_{a}(t,n)\cdot W_{r}\left[\tau -2R_{n}^{\prime }(t)/c\right]\cdot \exp\left[-j4\pi /\lambda \cdot R_{n}^{\prime }(t)\right]\\ & \cdot \exp\left\{-{\left(-1\right)}^{\left|n\right|}j\pi k_{r}\cdot {\left[\tau -2R_{n}^{\prime }(t)/c\right]}^{2}\right\} \end{array}$$

where *σ* is the backscattering intensity, *λ* is the wave length, *W_r_*(·) and *W_a_*(·) are the antenna patterns of range and azimuth direction. 

We can get the signals for main area as:

(4) $$\begin{array}{ll} s_{main}(\tau ,t) & =\sigma \cdot W_{a}(t)\cdot W_{r}\left[\tau -2R(t)/c\right]\cdot \exp\left[-j4\pi /\lambda \cdot R(t)\right]\\ & \cdot \exp\left\{-j\pi k_{r}\cdot {\left[\tau -2R(t)/c\right]}^{2}\right\} \end{array}$$

We can see from (3) and (4) that the ambiguous signals with odd *n* show contrary modulation rate to the main signals, whereas those with even *n* show the same modulation rate. 

The filtering of an up chirp with a down chirp filter will result in defocusing phenomenon along range direction and the pulse compression result as follow [12]:

(5) $$s_{de}(\tau )=1/\sqrt{2}\cdot rect\left[\tau /2\cdot T_{p}\right]\cdot \exp\left(-j\pi k_{r}\tau^{2}/2\right)$$

For comparison, the result of the correct filtering of the down chirp is [12]:

(6) $$s_{down}(\tau )=\exp\left(-j\pi /4\right)\cdot \sqrt{k_{r}T_{p}^{2}}\sin c\left[\pi k_{r}T_{p}\tau \right]$$

where *k_r_T_p_*^2^ is the compression gain.

We can see that the amplitude of mismatched up-chirp ambiguous signals (odd *n*) is lowered by $1/\sqrt{2\cdot k_{r}T_{p}^{2}}$.

The signal power of main area is unchangeable once the system antenna pattern and imaging targets are fixed, which leaves ambiguity suppression work to the reduction of ambiguous signal power. Due to the contrary modulation rate to the main signals with odd ambiguous number, the method of alternating up and down chirp modulation is provided with lower ambiguous signal power owing to the defocusing of mismatched range filter. Therefore, it can be widely adopted in the multi-polarization spaceborne SAR where a serious decline in the range-ambiguity-to-signal-ratio appears in cross polarization mode.

We can get the range-ambiguity-to-signal-ratio (RASR) as [18]:

(7) $$RASR=\frac{\sum_{n=-\infty }^{\infty }\int_{-T_{s}/2}^{T_{s}/2}G^{2}\left[\eta \left(t,n\right)\right]\cdot \sigma \left(t,n\right)\sin\left[\eta \left(t,n\right)\right]/R_{n}^{\prime 3}(t)dt}{\int_{-T_{s}/2}^{T_{s}/2}G^{2}\left[\eta \left(t\right)\right]\cdot \sigma \left(t\right)\sin\left[\eta \left(t\right)\right]/R^{3}(t)dt}$$

where *η* represents the incidence angle for a certain ambiguous number, *G*(·) represents the azimuth antenna patterns for a certain incidence angle, *T_s_* represents the data recording time.

As can be seen from (7), the ambiguous signal power is mainly determined by the backscattering intensity of the ambiguous area when the range antenna pattern has already been fixed according to the system design. At certain condition, range antenna pattern and the lower power of the defocusing signal can no longer suppress the ambiguous signal power being lower than the main area signal power, when the backscattering intensity of ambiguous area grows strong enough. This can result in ambiguous range defocusing targets appearing on the product image with a shape of line along range direction, having severe impact on visual effects and subsequent applications.

## 3. Ambiguity Suppression Method

**Figure 2:**
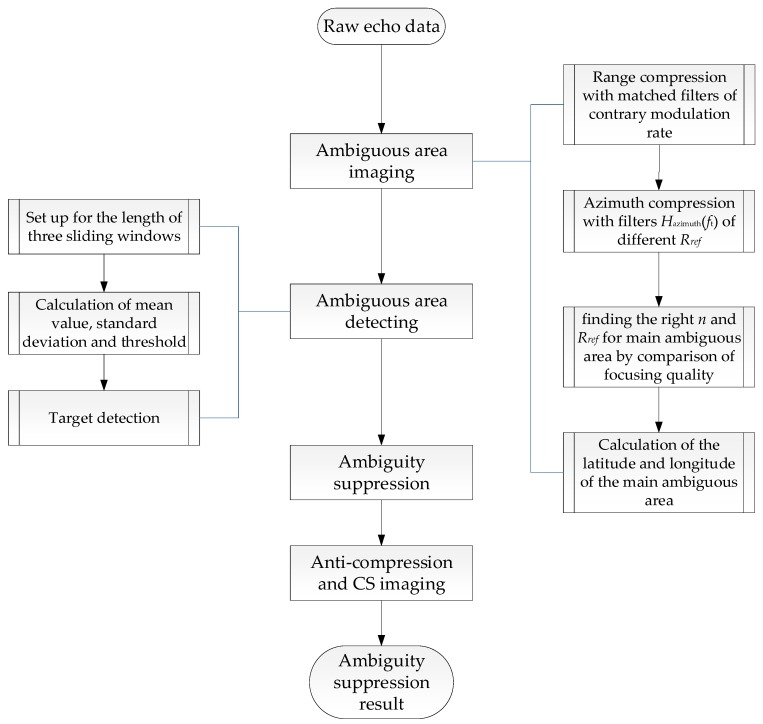
Figure which shows the detailed steps of the proposed range ambiguity suppression method.

In order to solve the problem of poor image quality caused by strong ambiguous signals, we developed an ambiguity suppression method aimed at ambiguous signals with odd ambiguous number. The general flowchart is given in Figure 2.

From previous analysis results we know that the ambiguous signals with odd *n* show contrary modulation rate to the main signals, whereas even *n* show the same rate. Therefore, we can get the image result of ambiguous area with odd *n* through range compression with contrary modulation rate to main signals and simple azimuth compression. Actually, SAR imaging can be regarded as a process to gather the scattered signal power again. Through ambiguous area imaging, we can get the focused ambiguous signal power rather than that spreads out in echo data. Then the focused ambiguous area can be detected and obtained by means of CFAR. Next, the ambiguity suppression is applied to the detected ambiguous area by simply reducing the amplitude, with no destruction to the phase information. Finally, echo data with suppressed ambiguous signals are obtained by anti-compression in range and azimuth direction. The image after ambiguity suppression are provided with Chirp-Scaling algorithm. The details of each step are as follows.

### 3.1. Ambiguous Area Imaging

The ambiguous area imaging method is based on a classical SAR imaging algorithm called Range-Doppler algorithm (RDA) [19]. In simple terms, RDA includes three steps which are range compression, range cell migration correction (RCMC) and azimuth compression, aiming at main area imaging. To get the image of the ambiguous area, we need to make some changes to the algorithm. The general steps are shown in the right parts of Figure 2. The following contents illustrate the specific process.

• Range compression

Firstly, we get the signal in range frequency domain by applying the fast Fourier transform (FFT) along range direction to the echo data as shown in Equations (1)–(4):

(8) $$\begin{array}{ll} S(f_{\tau },t,n) & =\sigma_{n}\cdot W_{a}(t,n)\cdot W_{r}\left[f_{\tau }\right]\cdot \exp\left[-j4\pi \left(f_{0}+f_{\tau }\right)/c\cdot R_{n}^{\prime }(t)\right]\\ & \cdot \exp\left[{\left(-1\right)}^{\left|n\right|}j\pi f_{\tau }^{2}/k_{r}\right] \end{array}$$

where *f_τ_* is the range frequency, *f*_0_ is the carrier frequency.

It is obvious that *n* = 0 presents the main area signals in range frequency domain and others the ambiguous area signals. 

A range matched filter with contrary modulation rate to the main area is required to get the pulse compression result of the ambiguous area. The filter in frequency domain is as follows [19]:

(9) $$H_{contrary}(f_{\tau })=\exp\left(j\pi f_{\tau }^{2}/k_{r}\right)$$

We can get the range compression result through the following matching filtering process:

(10) $$s_{r}(\tau ,t,n)=F^{-1}\left[S(f_{\tau },t,n)*H_{contrary}(f_{\tau })\right]$$

where *F*^−1^[·] represents the inverse FFT(IFFT). 

The specific expression is as follows:

(11) $$s_{odd}(\tau ,t,n)=\sigma_{n}\cdot W_{a}(t,n)\cdot \sin c\left[\tau -2R_{n}^{\prime }(t)/c\right]\cdot \exp\left[-j4\pi R_{n}^{\prime }(t)/\lambda \right]$$

(12) $$\begin{array}{ll} s_{even}(\tau ,t,n) & =\sigma_{n}\cdot W_{a}(t,n)\cdot W_{r}\left[\left(\tau -2R_{n}^{\prime }(t)/c\right)/2\right]\cdot \exp\left[-j4\pi R_{n}^{\prime }(t)/\lambda \right]\\ & \cdot \exp\left[-j\pi k_{r}/2\cdot {\left(\tau -2R_{n}^{\prime }(t)/c\right)}^{2}\right] \end{array}$$

where *s_odd_*(*τ*,*t*,*n*) represents the compression results of odd *n*, *s_even_*(*τ*,*t*,*n*) the even *n* (including main area with *n* = 0).

We can see that only the ambiguous area with odd *n* can obtain the right focused range profiles, contributing to the acquisition of the focused image of this ambiguous area. 

• Azimuth compression

There are two steps that need to be followed, including RCMC and azimuth compression, according to the RDA. However, the coupling of range and azimuth should remain unchanged to keep the imaging process reversible, which makes it impossible to add RCMC to the imaging process. Thus, we get the image result of ambiguous area through simple azimuth matched filter compression under the assumption of tiny range migration.

Here we consider *s_odd_*(*τ*,*t*,*n*) only since the defocusing range compression results can no longer get the focusing image through azimuth processing. We get the range compression results in Doppler frequency domain by azimuth FFT [19]:

(13) $$\begin{array}{ll} S_{rd\_odd}(\tau ,f_{t},n) & =\sigma_{n}\cdot W_{a}(f_{t},n)\cdot \sin c\left[\tau -2R_{n}^{\prime }(f_{t})/c\right]\\ & \cdot \exp\left[-j4\pi R_{0}/\lambda \cdot \left(\sqrt{1-{\left(\lambda f_{t}/2V\right)}^{2}}\right)\right] \end{array}$$

where *f_t_* is the azimuth frequency, *R*_0_ is the center slant range, *V* is the equivalent velocity between satellite and target at center time. *R^’^_n_*(*f_t_*) is the range cell migration in the range direction, which has a complex expression that can be found in [19].

The azimuth matched filter in frequency domain is as follows [19]:

(14) $$H_{azimuth}(f_{t})=\exp\left[j4\pi R_{ref}/\lambda \cdot \left(\sqrt{1-{\left(\lambda f_{t}/2V\right)}^{2}}-1\right)\right]$$

where *R_ref_* represents the reference slant range.

According to the SAR imaging algorithm, *R_ref_* and *V* are calculated in the standard imaging process apparently for main area signals [20]. Considering the long slant range of the spaceborne SAR, *V* of the ambiguous area varies little to the main area. Thus, we just need an appropriate *R_ref_* for the ambiguous area. According to the range ambiguity theory, it can be expressed as:

(15) $$R_{ref}=R_{main}+c/2\cdot n/prf$$

where *R_main_* represents the calculated reference slant range from standard imaging process.

We can get the ambiguous area image result of certain *n* through the following matching filtering process:

(16) $$s_{im}(\tau ,t,n)=F^{-1}\left\{\left[S_{rd\_odd}(\tau ,f_{t},n)+S_{rd\_even}(\tau ,f_{t},n)\right]*H_{azimuth}(f_{t})\right\}$$

where *S_rd_even_*(*τ*,*f_t_*,*n*) represents the range compression results of even *n* in Doppler frequency domain.

• Finding the main ambiguous area

For better ambiguity suppression, we need to find the right *n* and *R_ref_* for main ambiguous area which shows strongest intensity in the focused image. Considering the mismatched *R_ref_* leads to defocusing targets, we just need to test the quality of images acquired by different *R_ref_*(*n*) and select the best focusing one. Generally, the focusing quality of a SAR image can be measured by image entropy [21]:

(17) $$\begin{array}{cc} E_{im} & =-\sum_{x=1}^{M}\sum_{y=1}^{N}\frac{I\left(x,y\right)}{S}\log\frac{I\left(x,y\right)}{S}\\ S & =\sum_{x=1}^{M}\sum_{y=1}^{N}I\left(x,y\right) \end{array}$$

where *I*(*x*,*y*) represents the gray scale value of the SAR image, *M* and *N* represent the total number of sample points in azimuth and range direction. The smaller the image entropy, the better the focused image.

What needs to be specified is that larger *n* can bring about smaller power of the corresponding ambiguous area as a result of farther distance from the antenna center. Thus, in this paper we choose *n* = 1 and *n* = −1 to find the location of the main ambiguous area.

• Main ambiguous area location

The right *n* can not only bring right focused main ambiguous area image, but also the center longitude and latitude of the main ambiguous area.

Supposing ***R***(*R_x_*,*R_y_*,*R_z_*) represents the center position coordinates of the main ambiguous area in the earth coordinate system, *α* and *β* represent the longitude (positive the East while negative the West) and latitude (positive the North while negative the South) respectively, we can get the following expression:

(18) $$\begin{array}{l} R_{x}=R_{T}\cdot \cos\alpha \cdot \cos\beta \\ R_{y}=R_{T}\cdot \cos\alpha \cdot \sin\beta \\ R_{z}=R_{T}\cdot \sin\alpha \end{array}$$

where *R_T_* represents the Earth radius at the location of the imaging target.

According to the geometric relationship of the satellite and Earth, we can get the following equations [22]:

(19) $$\begin{array}{c} \left|R_{s}-R\right|={R^{\prime }}_{cen}\\ \frac{V_{s}\times \left(R_{s}-R\right)}{{R^{\prime }}_{cen}}=\frac{\lambda f_{dc}}{2}\\ \frac{\left(R_{x}^{2}+R_{y}^{2}\right)}{R_{equ}}+\frac{R_{z}^{2}}{R_{pol}}=1 \end{array}$$

where ***R_s_*** and ***V_s_*** represent the position and velocity vector of satellite at the center time, *f_dc_* the Doppler center frequency, *R_equ_* and *R_pol_* the radius of the equator and pole of the Earth. *R’_cen_* represents the center slant range of main ambiguous area that can be obtained by:

(20) $${cen}_{R}^{\prime }=R_{cen}+n/prf\cdot c/2$$

where *R_cen_* represents the center slant range of main imaging area.

By solving the Equations (18)–(20), we can get the value of *α* and *β.* The optical image can be obtained by the information of longitude and latitude, which can be compared with the acquired SAR image to verify the validity of the imaging process.

### 3.2. Ambiguous Area Detecting Based on CFAR

The imaging result of main ambiguous area includes not only ambiguous targets but also range defocusing results of the main area and other ambiguous areas which show weaker backscattering intensity than focusing ones, bringing about even weaker intensity than the focusing ambiguous targets. The problem of ambiguous area target detection in the background of defocusing targets can be translated into the detection for strong backscattering targets under weak background, which is normally solved by a target detecting algorithm called CFAR [23,24].

Target detection based on CFAR is an algorithm that requires a given detection rate and a strong contrast between target and background, which is extensively used in ship detection of SAR image [25,26]. The excellent results of ship detection demonstrate the ability of CFAR in strong scatter points detecting under weak background of SAR images, which is what we need for ambiguous area detection in this paper.

**Figure 3:**
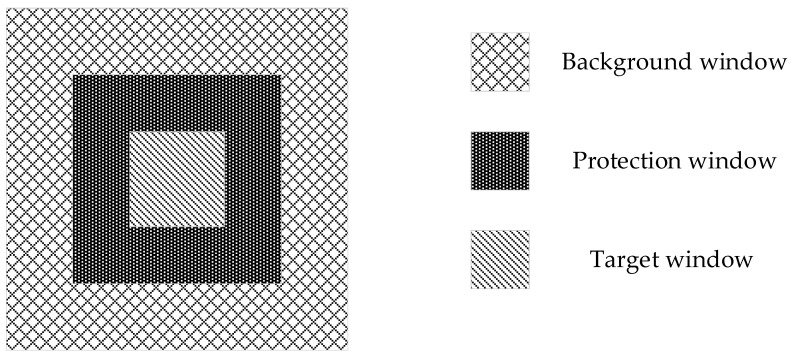
This is a figure that shows the geometric relation of three sliding windows. They are all squares and the inside is the target window, the middle is the protection window, and the outside is the background window.

In this paper we choose the double-parameter CFAR detection algorithm to achieve the detection of main ambiguous area targets [25,26,27,28]. This algorithm sets up three sliding windows as shown in Figure 3 and realize the target detection by comparison of the pixel grayscale in target window and the self-adapting threshold which is acquired by false alarm rate (*PFA*) and background clutter modeling, which is assumed to be Gaussian distribution with mean value *μ* and standard deviation *σ* in background window. The general flowchart is given in the left part of Figure 2. The specific steps are as follows.

• Set up for the length of sliding windows 

The size of the general target window should be the same as the minimum size of targets to be detected. The length of the protection window is generally twice the maximum target size, while the background window being selected as two or four times the protection window size. The step length of the sliding window is in accordance with the size of the target window. The ambiguous area image is usually an integrated area composed of strong scattering points, different from ships which have a uniform shape, making it necessary to do some experiments to determine the reasonable length of the three windows.

• Calculation of threshold including *μ* and *σ* for the background window

To set the detection threshold *T*, we need the distribution function of background clutter and the given *PFA*. Supposing *Φ*(*·*) presents the Gaussian distribution function, we can get the following expression:

(21) $$\begin{array}{l} T=\sigma \cdot t_{1}+\mu \\ t_{1}=\Phi^{-1}\left(1-PFA\right) \end{array}$$

where *μ* and *σ* are calculated according to each background window, making the threshold *T* changing adaptively. In fact, the background clutter may not be in conformity with the standard Gaussian distribution absolutely, forcing us to determine the reasonable choice of *t*_1_ through experiments.

• Target detection

If the gray value of a pixel in the target window is *I_tar_*, then the target detection criterion is:

(22) $$I_{tar}>\sigma \cdot t_{1}+\mu$$

As mentioned above, we need some experiments to determine the reasonable parameters of the algorithm. Limited to the characteristics of the ambiguous area image itself, this method cannot evaluate the detection quality by the specific quantity of right detecting targets. We can only find a comparatively good value interval by comparing the results from visual observation.

### 3.3. Ambiguity Suppression

The phase information of the input data should stay complete and not damaged to keep the integrity of main area signals, which makes it necessary to the use of amplitude suppression for ambiguity suppression. We divided the image result acquired in Section 3.1 into two parts according to the detection result:

(23) $$s_{im}(x,y)=\sum_{i,j\in C_{re}}s_{im}(i,j)+\sum_{p,q\in C_{\mathrm{de}}}s_{im}(p,q)$$

where *C_de_* represents the collection of detected pixel positions and *C_re_* represents the collection of remaining pixel positions.

The amplitude of detected pixel is reduced by *N* times, and we can get the result as:

(24) $$s_{pr}(x,y)=\sum_{i,j\in C_{re}}s_{im}(i,j)+\sum_{p,q\in C_{\mathrm{de}}}s_{im}(p,q)/N$$

The extent of suppression is alternative for manually input. Generally, we choose the attenuation multiplier *N* for the aim that ambiguous signal power lower than main area power after standard imaging by 0~5 dB.

### 3.4. Anti-Compression and Imaging

Since the imaging and ambiguity suppression process are reversible, echo data can be obtained by anti-compression in range and azimuth direction to the data after ambiguity suppression. The image after ambiguity suppression are provided with Chirp-Scaling algorithm.

The proposed ambiguity suppression method has two limitations:

- Only for ambiguous areas with odd ambiguous numbers;
- Only for strip data with tiny range migration.

## 4. Results

**Figure 4:**
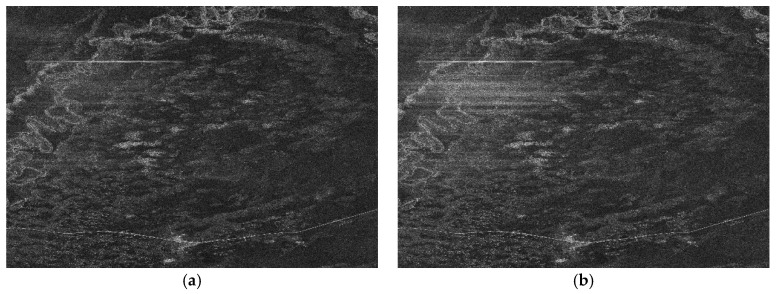
GF-3 satellite images in full-polarization strip mode which acquired in the near Argun River of Inner Mongolia. (**a**) is the HH-polarization mode, (**b**) is the VV-polarization mode.

The performance of the proposed range ambiguity suppression method has been analyzed by processing full-polarization SAR data from Gaofen-3 sensor strip mode which acquired in the near Argun River of Inner Mongolia (the parameters are listed Table 1). Figure 4 shows the HH polarization and VV polarization SAR images acquired by applying standard Chirp-Scaling algorithm to the echo data with no ambiguity suppression process. There are some obvious bright lines along range direction in the top left of the two images which have serious implications on the visual effect and subsequent applications. Considering the technology of alternatively up and down chirp modulation implemented by Gaofen-3 satellite, the bright lines in the image could be the defocusing range ambiguous signals which hold strong intensity.

**Figure 5:**
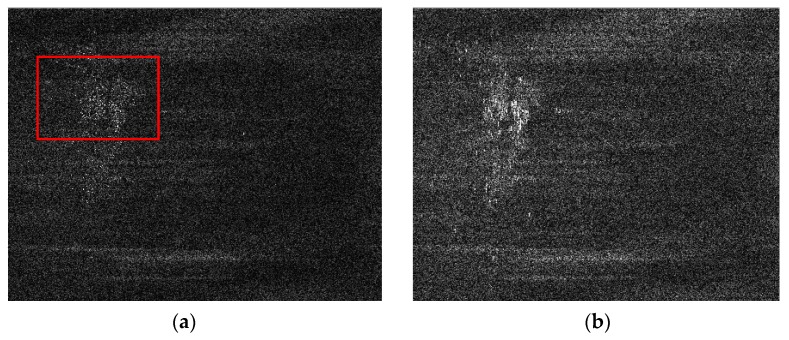
Image results of the ambiguous area: (**a**) represents the azimuth matched filter *R_ref_* with *n* = −1; (**b**) represents the azimuth matched filter *R_ref_* with *n* = 1.

Figure 5 shows the image of ambiguous area targets by processing the HH polarization echo data, where (**a**) represents the azimuth matched filter *R_ref_* with *n* = −1 when (**b**) represents the *n* = +1. To find the right *n* of the main ambiguous area, entropy values of the two are calculated according to Equation (17). The results are as follows:

(25) $$\begin{array}{l} E_{n=1}=2.8438\\ E_{n=-1}=0.1278 \end{array}$$

It’s obvious that the focusing performance of the result with n = −1 is better than that with n = +1, with which we can suppose that the main ambiguous area of the bright lines is with *n* = −1. The center latitude and longitude information can be calculated according to the Equations (18)–(20) and Table 1.

(26) $$\begin{array}{l} Lon=120.921{}^{\circ}E\\ Lat=48.833{}^{\circ}N \end{array}$$

**Figure 6:**
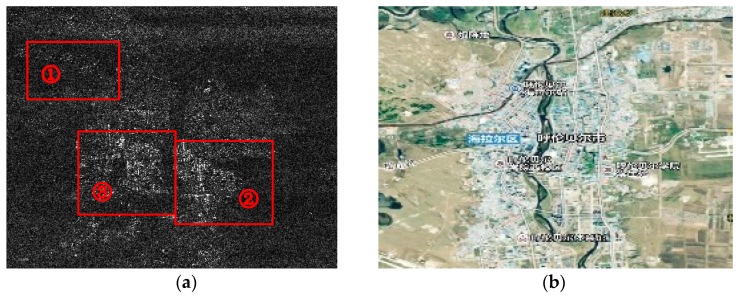
(**a**) magnification details of the main ambiguous area image with *n* = −1 shown in Figure 5a; (**b**) shows the corresponding optical image obtained by latitude and longitude information of *n* = −1.

Figure 6b shows the optical image of the main ambiguous area with the result above. It is the city of Hulun-Buir near the Argun River, which displays a stronger backscatter intensity than the imaging area (Argun River) indeed. Figure 6a shows the corresponding SAR image which is the detail magnification of the top left part in Figure 5a (marked with a red rectangle). The two show a high degree of consistency, which can verify the validity of the ambiguous area imaging process.

**Figure 7:**
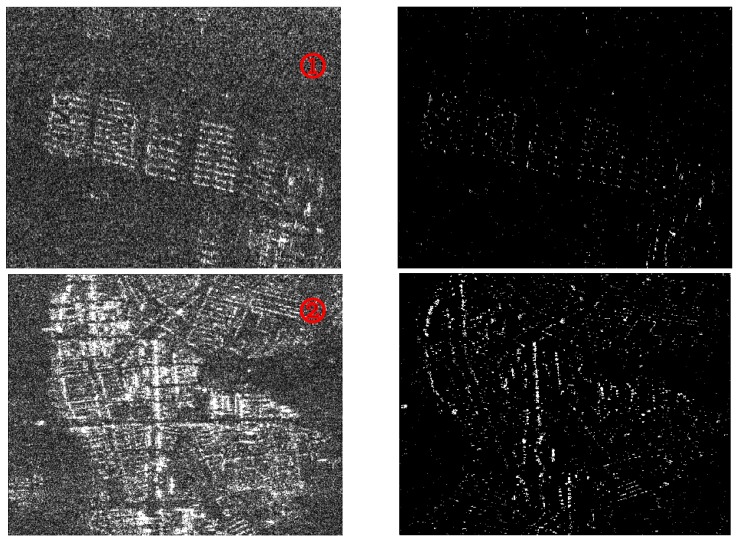

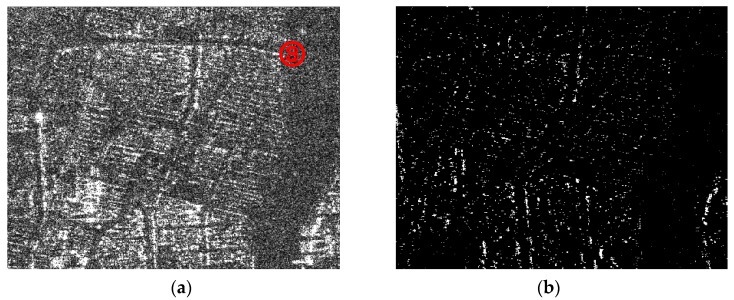
Detection results of ambiguous area targets based on CFAR. (**a**) represents the ambiguous area imaging results; (**b**) represents the detection results.

The parameters of CFAR used in target detection process are shown in Table 2. We set up the target window as 2, the protection window as 8 and the background window as 32, and we finally choose *t*_1_ = 2.5~3.5 for the calculation of threshold, which is decided after many times of experiments. In fact, it can reach almost the same detection rate with few error targets for *t*_1_ in this interval. Figure 7 shows the detecting results of ambiguous area targets where left parts present ambiguous area imaging results which are typical parts of Figure 6a (marked with red rectangles with serial numbers), right parts the detecting results with *t*_1_ = 3. It can be seen from the results that most of the strong scatters in imaging results are detected which presents the composition of the ambiguous area. The results show the accuracy and validity of the detection algorithm.

We selected an attenuation multiplier *N* = 100 according to the power of the ambiguous area and main imaging area. Actually, the performance of ambiguity suppression depends on the detection results more than the attenuation multiplier *N*. Thus, there is no need to be entangled in the precise choice of *N* as long as it can suppress the ambiguous power effectively.

**Figure 8:**
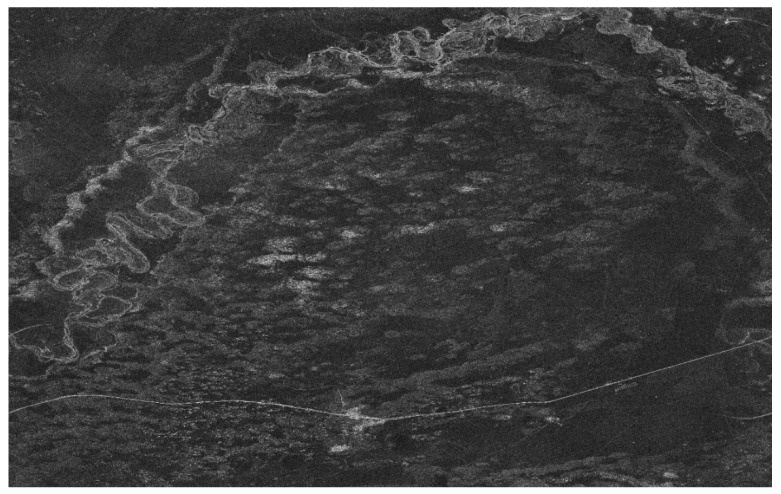
The range ambiguity suppression result of HH-polarization mode.

**Figure 9:**
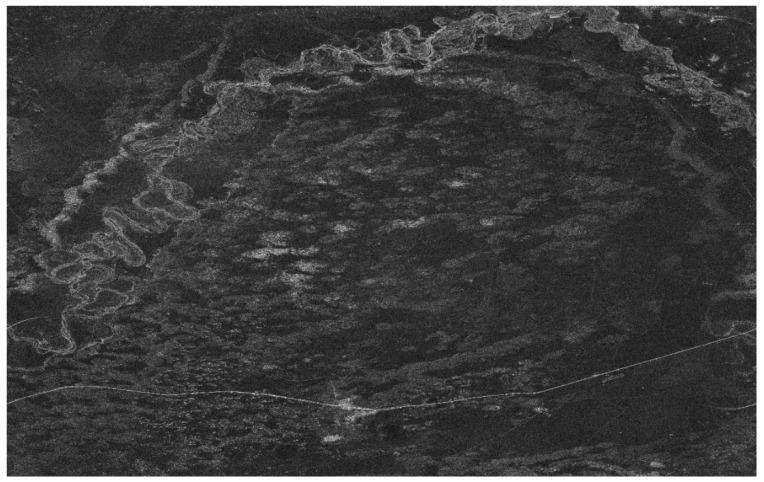
The range ambiguity suppression result of VV-polarization mode.

The image results after ambiguity suppression and chirp-scaling algorithm imaging are shown in Figure 8 (HH-polarization) and Figure 9 (VV-polarization). Comparing with the results in Figure 4a,b, respectively, the bright lines in the top left part of the image are almost invisible. It indicates that the ambiguity signal power in data have been well suppressed and the effect of ambiguity suppression is remarkable. The results show the accuracy and validity of the proposed ambiguity suppression algorithm.

## 5. Conclusions

In this paper, we have proposed a new range ambiguity suppression method for up and down chirp modulation. The most important feature is the ambiguous target detection based on pulse compression using a contrary modulation rate and CFAR detection method. The method can obtain the ambiguity area image and reduce the ambiguity signal power appropriately. The effectiveness and correctness of the approach are demonstrated by processing archive images acquired by the Gaofen-3 SAR sensor in full-polarization mode. Therefore, the proposed method can be viewed as an alternative way to improve the image quality for images with range ambiguity. At the same time, the method also has some limitations as mentioned in Section 3.4, and these limitations along with the problem of scientifically evaluating the detection results are the direction of the research in the future.

## Figures and Tables

**Table 1:** GF-3 Satellite parameters.

Radar Parameter	Value	Radar Parameter	Value
λ	0.055517 m	PRF	1292.0768
Transmit Band	40 MHz	Look angle	38.91°
Sample rate	66.667 MHz	Incidence angle	44.64°
Modulation rate	1.6006 × 10^12^	Near range	1001.7 km
Satellite velocity	(−1677.18, 5525.42, −4885.91)	Reference range	1015.3 km
Satellite position	(−2,870,758.09, 3,815,169.12, 5,287,687.27)	Center range	1015.3 km
Target position	118.4872° E, 49.2979° N	Target radius	6,368,250 m
Equatorial radius	6,378,140 m	Polar radius	6,356,755 m
equivalent velocity	7097.4 m/s	Fdc	6.508994

**Table 2:** Parameters used in CFAR.

Parameter	Value
Target window length	2
Protection window length	8
Background window length	32
*t*_1_	2.5~3.5
